# Identification and validation of necroptosis-related prognostic gene signature and tumor immune microenvironment infiltration characterization in esophageal carcinoma

**DOI:** 10.1186/s12876-022-02423-6

**Published:** 2022-07-15

**Authors:** Kai Sun, Juan-juan Hong, Dong-mei Chen, Zhan-xiong Luo, Jing-zhang Li

**Affiliations:** 1 Department of Oncology, Liuzhou People’s Hospital, Liuzhou, 545001 Guangxi Zhuang Autonomous Region China; 2 Guilin Medical University, Guilin, 541010 Guangxi Zhuang Autonomous Region China

**Keywords:** Esophageal carcinoma, Necroptosis, Prognosis, Signature, Immune infiltration

## Abstract

**Background:**

Esophageal carcinoma (ESCA) is a common malignancy with a poor prognosis. Previous research has suggested that necroptosis is involved in anti-tumor immunity and promotes oncogenesis and cancer metastasis, which in turn affects tumor prognosis. However, the role of necroptosis in ESCA is unclear. This study aimed to investigate the relationships between necroptosis-related genes (NRGs) and ESCA.

**Methods and results:**

The clinical data and gene expression profiles of ESCA patients were extracted from The Cancer Genome Atlas (TCGA), and 159 NRGs were screened from the Kyoto Encyclopedia of Genes and Genomes (KEGG) database. We then identified 52 differentially expressed NRGs associated with ESCA and used them for further analysis. Gene ontology (GO) and KEGG functional enrichment analyses showed that these NRGs were mostly associated with the regulation of necroptosis, Influenza A, apoptosis, NOD-like receptor, and NF-Kappa B signaling pathway. Next, univariate and multivariate Cox regression and LASSO analysis were used to identify the correlation between NRGs and the prognosis of ESCA. We constructed a prognostic model to predict the prognosis of ESCA based on *SLC25A5, PPIA*, and *TNFRSF10B*; the model classified patients into high- and low-risk subgroups based on the patient’s risk score. Furthermore, the receiver operating characteristic (ROC) curve was plotted, and the model was affirmed to perform moderately well for prognostic predictions. In addition, Gene Expression Omnibus (GEO) datasets were selected to validate the applicability and prognostic value of our predictive model. Based on different clinical variables, we compared the risk scores between the subgroups of different clinical features. We also analyzed the predictive value of this model for drug sensitivity. Moreover, Immunohistochemical (IHC) validation experiments explored that these three NRGs were expressed significantly higher in ESCA tissues than in adjacent non-tumor tissues. In addition, a significant correlation was observed between the three NRGs and immune-cell infiltration and immune checkpoints in ESCA.

**Conclusions:**

In summary, we successfully constructed and validated a novel necroptosis-related signature containing three genes (*SLC25A5, PPIA,* and *TNFRSF10B*) for predicting prognosis in patients with ESCA; these three genes might also play a crucial role in the progression and immune microenvironment of ESCA.

**Supplementary Information:**

The online version contains supplementary material available at 10.1186/s12876-022-02423-6.

## Introduction

An estimated 604,100 new cases of esophageal cancers (ESCA) were diagnosed, and 544,076 related deaths were observed worldwide in 2020; furthermore, ESCA ranks seventh most commonly diagnosed cancer and sixth in cancer-related mortality [1]. Despite great curative progress in surgical treatment, chemoradiotherapy and systemic treatment, which has largely improved the survival rate of tumor patients, the prognosis of ESCA remains poor [2]. This to a large extent is because the condition is mostly diagnosed at an advanced stage and symptoms are non-specific in the early stages, with the 5-year survival rate being lower than 15% [3]. Thus, there is a need to identify more predictive and diagnostic tumor biomarkers to achieve a good outcome in ESCA patients. Therefore, finding more marker-related genes predicting the prognosis of patients could be an important strategy for the treatment of ESCA.

Currently, most anticancer drugs mainly trigger cancer cell death by induction of apoptosis [4]. However, apoptosis resistance makes cancer cells drug-resistant and limits the efficacy of therapies. With the in-depth study of the mechanism of cell death, more cell death forms are being continuously discovered. Necroptosis, a newly recognized mechanism of programmed necrosis, has similar characteristics to both necrosis and apoptosis [5]. Morphological features of necroptosis include cell and organelle swelling, plasma membrane rupture, and organelle breakdown, leading to the leakage of intracellular contents and subsequently inducing an inflammatory response [6]. Necroptosis is triggered through the activation of cellular receptors, mainly including tumor necrosis receptor (TNFR1), death receptors (Fas/FasL), Toll-like receptors (TLRs), and cytosolic nucleic acid sensors [7]. Necroptosis generally relies on the kinase activity of receptor interacting proteins (RIPs) [8]. Furthermore, the RIPK1/RIPK3/MLKL pathway appears to be a key pathway mediating necroptosis.

There exists a complex relationship between necroptosis and cancer. Necroptosis affects tumorigenesis, infiltration, and metastasis, thereby affecting cancer prognosis. Accumulating evidence suggested that necroptosis not only mediates physiological regulation but also results in inflammatory pathologies and various cancers such as gastric cancer, colorectal cancer, cholangiocarcinoma, pancreatic cancer, and hepatocellular carcinoma [9–13]. ZENG et al. [14] confirmed that RIPK1 expression level was significantly upregulated in colorectal adenocarcinoma samples, while that of RIPK3 and p-MLKL were downregulated, suggesting that these cancer cells escape necroptosis for their survival. In cholangiocarcinoma, matrine induces necroptosis by enhancing RIP3 expression, producing reactive oxygen species (ROS), and activating the downstream RIP3/MLKL/ROS signaling pathway [15]. Furthermore, apoptosis-resistant tumors have been shown to be regressed by inducing necroptosis [16]. Necroptosis, especially necroptosis-related genes (NRGs), plays an important role in cancer. However, to date, the role of NRGs in ESCA remains unclear. This study, therefore, investigates the relationship between NRGs and ESCA to contribute to the diagnosis and prognosis of the disease.

In this study, we used the TCGA database to explore the relevance of NRGs in predicting the prognosis of ESCA. We thus constructed a necroptosis risk-scoring prognosis signature based on the screened prognostic NRGs. Subsequently, the applicability and prognostic value of the predictive model were validated using a GEO ESCA database. We also investigated the expression levels of the candidate NRGs in 20 ESCA tissues and matched adjacent normal tissues by immunohistochemistry (IHC). In addition, we examined the relationship between NRGs and the immune microenvironment in ESCA. This study may provide additional information regarding diagnosis and prognostic biomarkers for ESCA.

## Results

### Expression levels of NRGs in ESCA

**Fig. 1:**
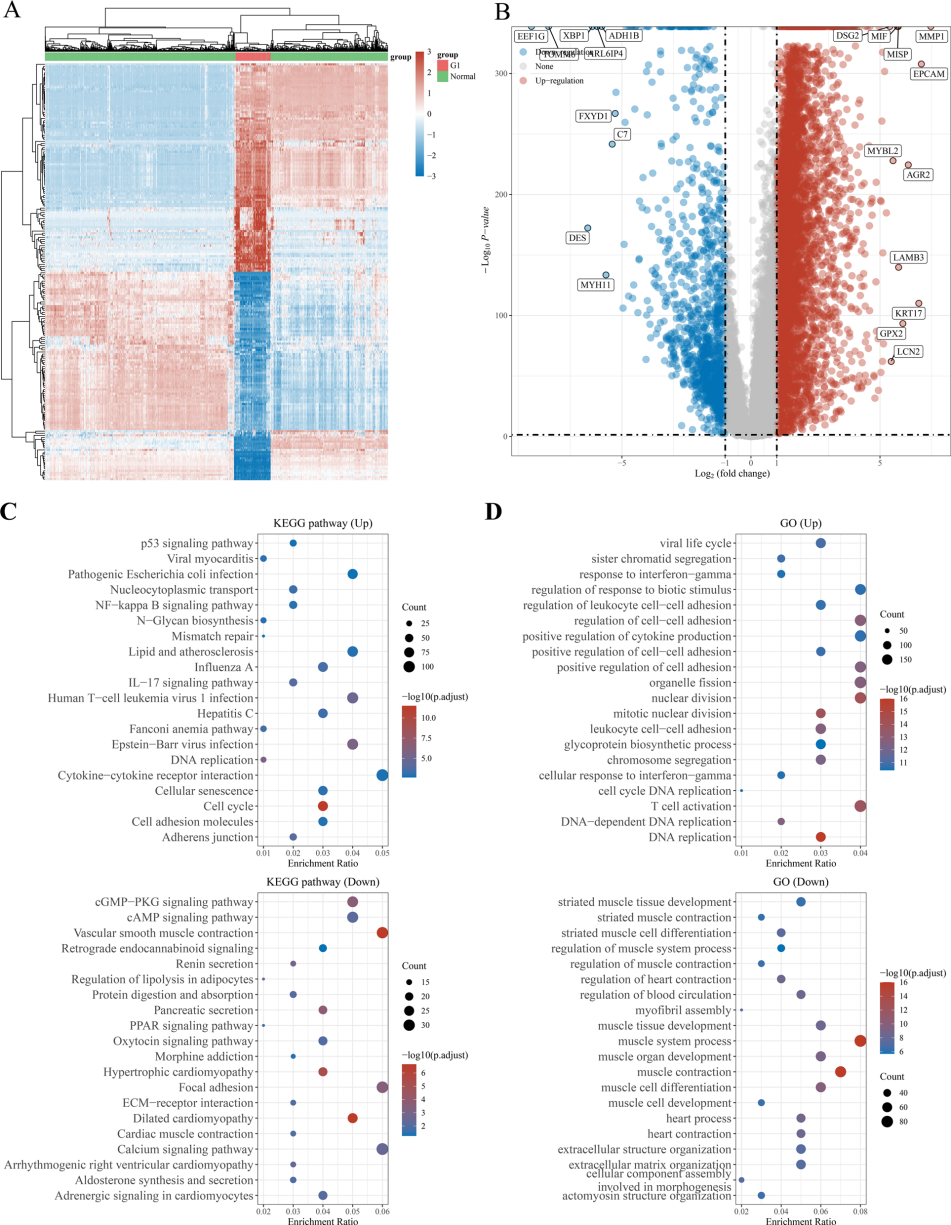
Differentially expressed NRGs in ESCA. **A** Heatmap of the 6000 differentially expressed DEGs in EC. **B** volcano plot of 6000 differentially expressed DEGs in ESCA. **C**, **D** Enriched Gene Ontology terms and KEGG pathways associated with the 6000 DEGs in ESCA. DEGs, differentially expressed genes; ESCA, Esophageal carcinoma; KEGG, Kyoto Encyclopedia of Genes and Genomes; GO: Gene ontology

**Fig. 2:**
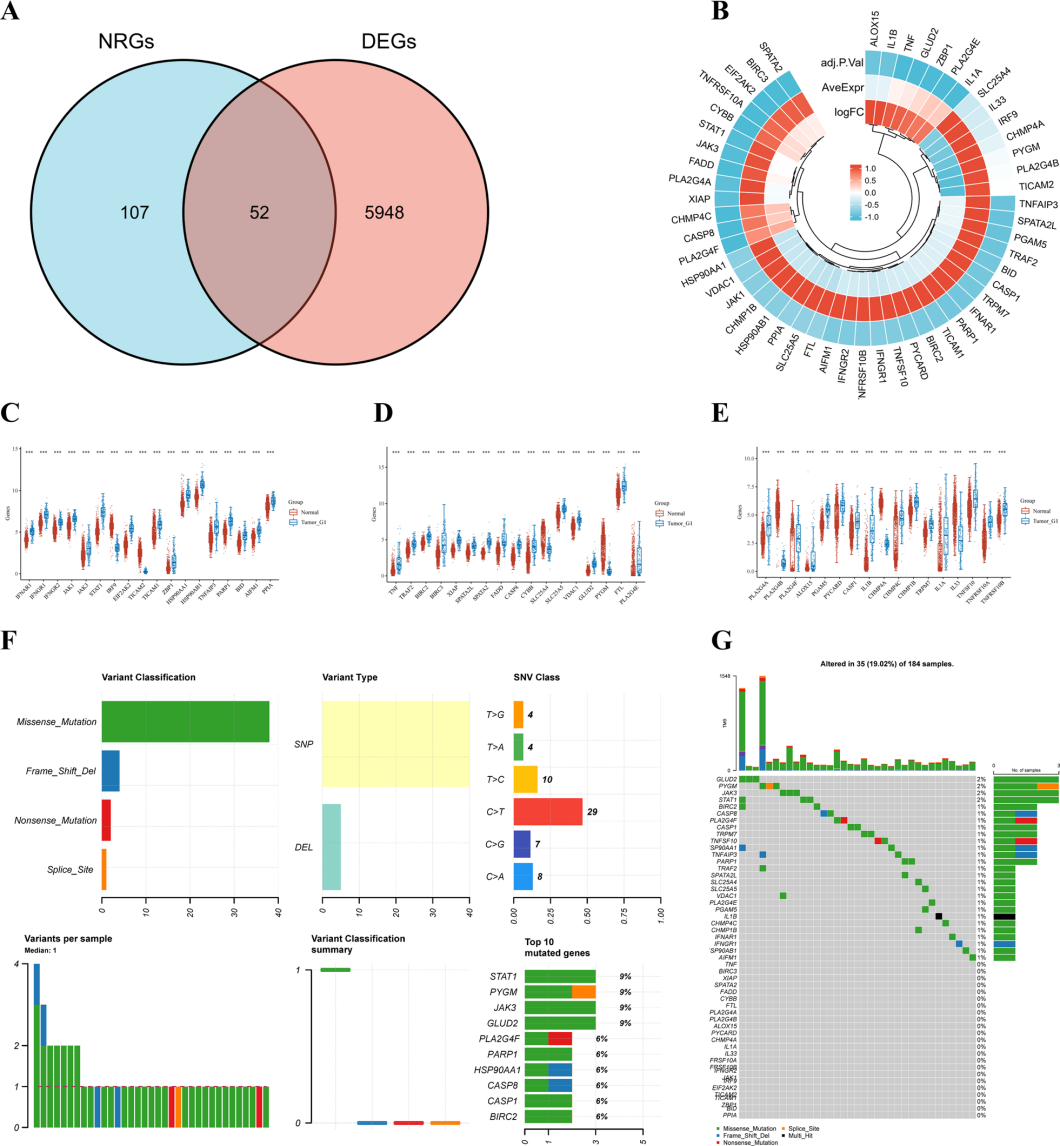
Landscape of NRGs expression and mutation. **A** Venn diagram of the intersection of NRGs and DEGs. **B** A total of 52 NRGs among the DEGs between ESCA and normal samples. **C**, **D**, **E** The expression of 52 NRGs in ESCA and normal samples, Normal, red; Tumor, blue. **F**, **G** The mutation frequency and classification of 52 NRGs in ESCA. DEGs, differentially expressed genes; NRGs, necroptosis-related genes; ESCA, Esophageal carcinoma

We acquired 6000 differentially expressed genes (DEGs) between 162 ESCA and 1456 normal tissues obtained from the UCSC Xena database and drew a heat map based on the expression level of each gene (Fig. 1A). Of these 6000 DEGs, 4820 were upregulated and 1180 were downregulated, as revealed by a volcano plot (Fig. 1B). The Gene ontology (GO) functional enrichment and Kyoto Encyclopedia of Genes and Genomes (KEGG) pathway analyses were summarized to clarify the biological significance of DEGs (Fig. 1C, D). The cut-off criteria for DEG were |Log2-fold change |> 1 and adjusted P-values < 0.05. We retrieved 159 NRGs from KEGG (Additional file 1: Table S1), which were further analyzed. Of these 159, 52 NRGs were identified among the 6000 DEGs (Additional file 2: Table S2, Fig. 2A, B). More specifically, of the 52 NRGs that were differentially expressed, 45 were upregulated and 7 were downregulated in ESCA samples compared with their expression in normal samples (Fig. 2C–E).

### Genetic variation and expression of NRGs

To evaluate the role of NRGs in tumorigenesis, we summarized the mutation frequency and variant classification of the 52 NRGs screened in ESCA samples. As shown in Fig. 2F, G, genetic mutations of NRGs were observed in 19.02% (35 / 184) of the ESCA samples. The most common of all mutations were missense mutations (Fig. 2F). SNPs were the most common type of variation, and C > T ranked as the highest SNV class. Among the 52 NRGs, *GLUD2, PYGM, JAK3,* and *STAT1* were the genes with the highest mutation frequency (Fig. 2F, G).

### Functional enrichment analysis of NRGs

**Fig. 3:**
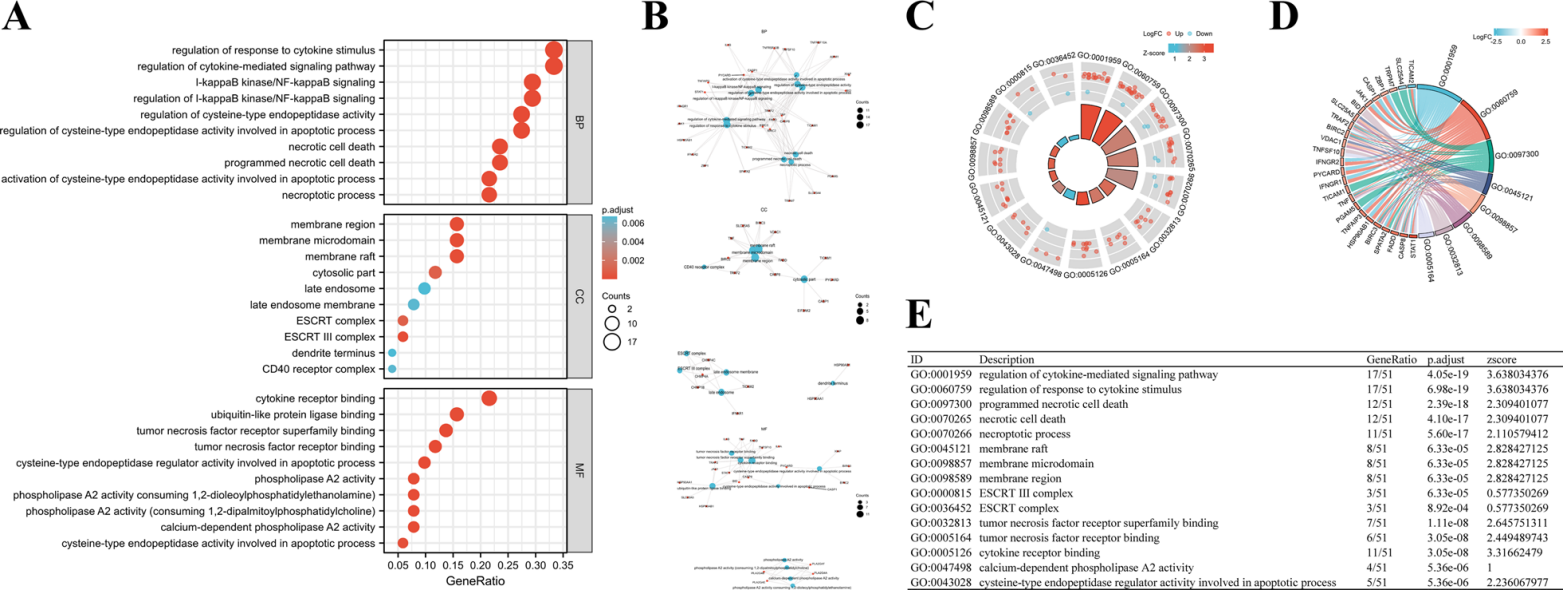
GO analysis of differentially expressed NRGs in ESCA. **A** The significant terms of GO function enrichment. **B** Network diagram of NRGs, blue nodes represent items, red nodes represent molecules, and the lines represent the relationship between items and molecules. **C** The GO circle shows scatter map of the specified gene’s logFC. **D** Enrichment string diagrams of NRGs. **E** Enrichment analysis network diagram, description of pathways. GO, Gene ontology; BP, biological process; CC cellular component; MF, molecular function; NRGs, necroptosis-related genes; ESCA, Esophageal carcinoma

**Fig. 4:**
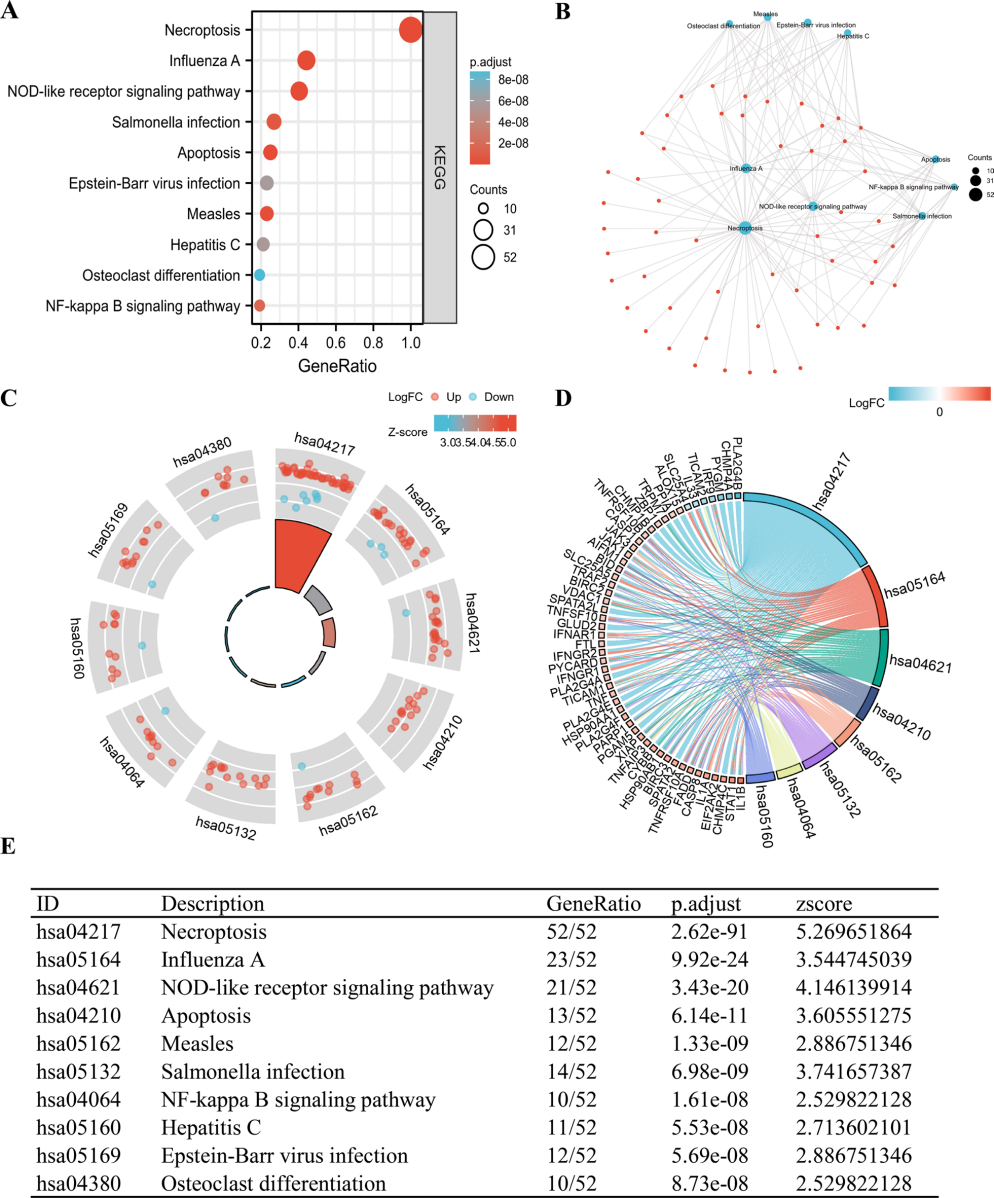
KEGG analysis of differentially expressed NRGs in ESCA. **A** The significant terms of KEGG analysis. **B** Network diagram of NRGs, blue nodes represent items, red nodes represent molecules, and the lines represent the relationship between items and molecules **C** KEGG circle shows scatter map of the specified gene’s logFC. **D** Enrichment string diagrams of NRGs.** E** Enrichment analysis network diagram, description of pathways. KEGG, Kyoto Encyclopedia of Genes and Genomes; NRGs, necroptosis-related genes; ESCA, Esophageal carcinoma

The GO functional enrichment and KEGG pathway analyses were performed to clarify the biological significance of NRGs. The enrichment analysis results of 52 NRGs are summarized in Figs. 3 and 4. GO terms are grouped into three major categories: biological processes (BP), cellular component (CC), and molecular function (MF). We found that the most significantly enriched BP terms were regulation of response to cytokine stimulus, regulation of cytokine-mediated signaling pathway, l-kappaB kinase/NF-kappaB signaling, cysteine-type endopeptidase activity, apoptotic process, and necrotic cell death; enriched CC terms were membrane region, membrane raft, membrane microdomain, cytosolic part, and ESCRT III complex; and enriched MF terms were cytokine receptor binding, ubiquitin-like protein ligase binding, tumor necrosis factor receptor superfamily binding, tumor necrosis factor receptor binding, cytokine receptor binding, and cysteine-type endopeptidase regulator activity involved in the apoptotic process (Fig. 3A). In terms of KEGG pathway analysis, these NRGs were mainly associated with pathways related to necroptosis, influenza A, NOD-like receptor signaling pathways, apoptosis, and Measles (Fig. 4A). Moreover, a network diagram of NRGs was plotted to represent the relationship between items and molecules (Figs. 3B, 4B). We then drew a circle of enriched GO and KEGG terms and combined them with Z-Score to predict the function of 52 NRGs in these pathways (Figs. 3C–E, 4C–E).

### Establishment of a necroptosis-related prognostic gene model for ESCA

**Fig. 5:**
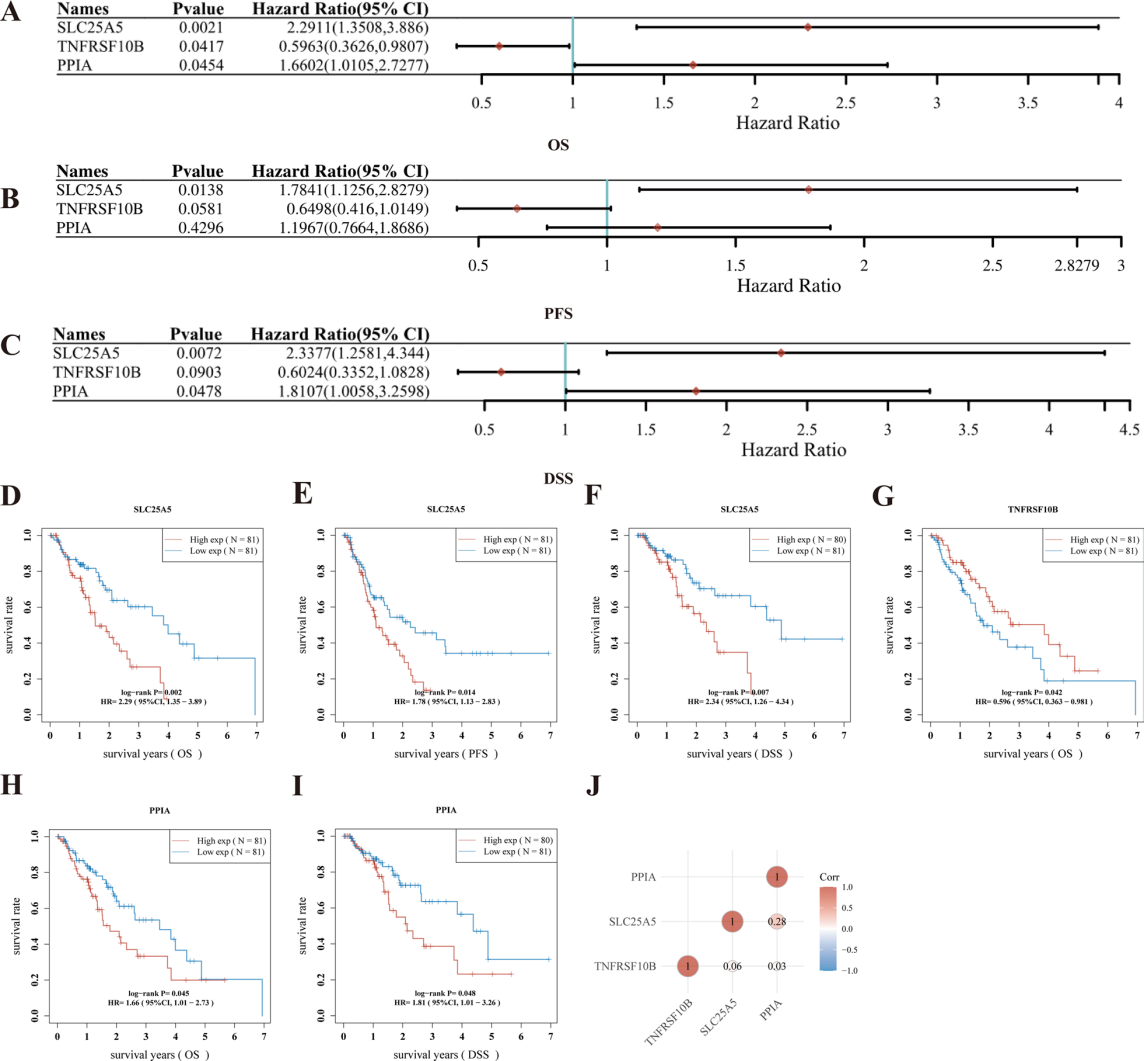
The prognostic value of NRGs in ESCA. **A**–**C** The overall survival curve, progression free survival, disease special survival of NRGs in ESCA. **D**–**F** The OS, PFS and DSS curve of SLC25A5 in ESCA. **G** The OS curve of TNFRSF10B in ESCA. **H**, **I** The DSS and OS curve of PPIA in ESCA patients. **J** Correlation analysis between three NRGs. ESCA, Esophageal carcinoma; OS, overall survival; PFS, progression-free survival; DSS, disease- specific Survival

To establish a prognostic risk signature, we performed a univariate Cox regression analysis of the 52 screened NRGs. As a result, three NRGs, namely *SLC25A5, PPIA,* and *TNFRSF10B*, were identified as genes with significant prognostic value in ESCA (Fig. 5A–C, Additional file 3: Fig. S1). The results of the Kaplan–Meier survival (KM) analysis (Fig. 5) suggested that high expression of *SLC25A5* was correlated with worse prognosis in ESCA patients (overall survival (OS), *p* = 0.002; progression-free survival (PFS), *p* = 0.014; disease-specific survival (DSS), *p* = 0.007) (Fig. 5D–F). Moreover, *PPIA high* expression was also associated with poor prognosis in ESCA patients (OS, *p* = 0.045; DSS, *p* = 0.048) (Fig. 5H, I). However, high *TNFRSF10B* expression resulted in a better prognosis in ESCA (OS, *p* = 0.042) (Fig. 5G). Furthermore, correlation analyses showed that *PPIA* was positively correlated with *SLC25A5* (Fig. 5J). The effect of interaction between *PPIA* and *SLC25A5* may be worthy of further study.

**Fig. 6:**
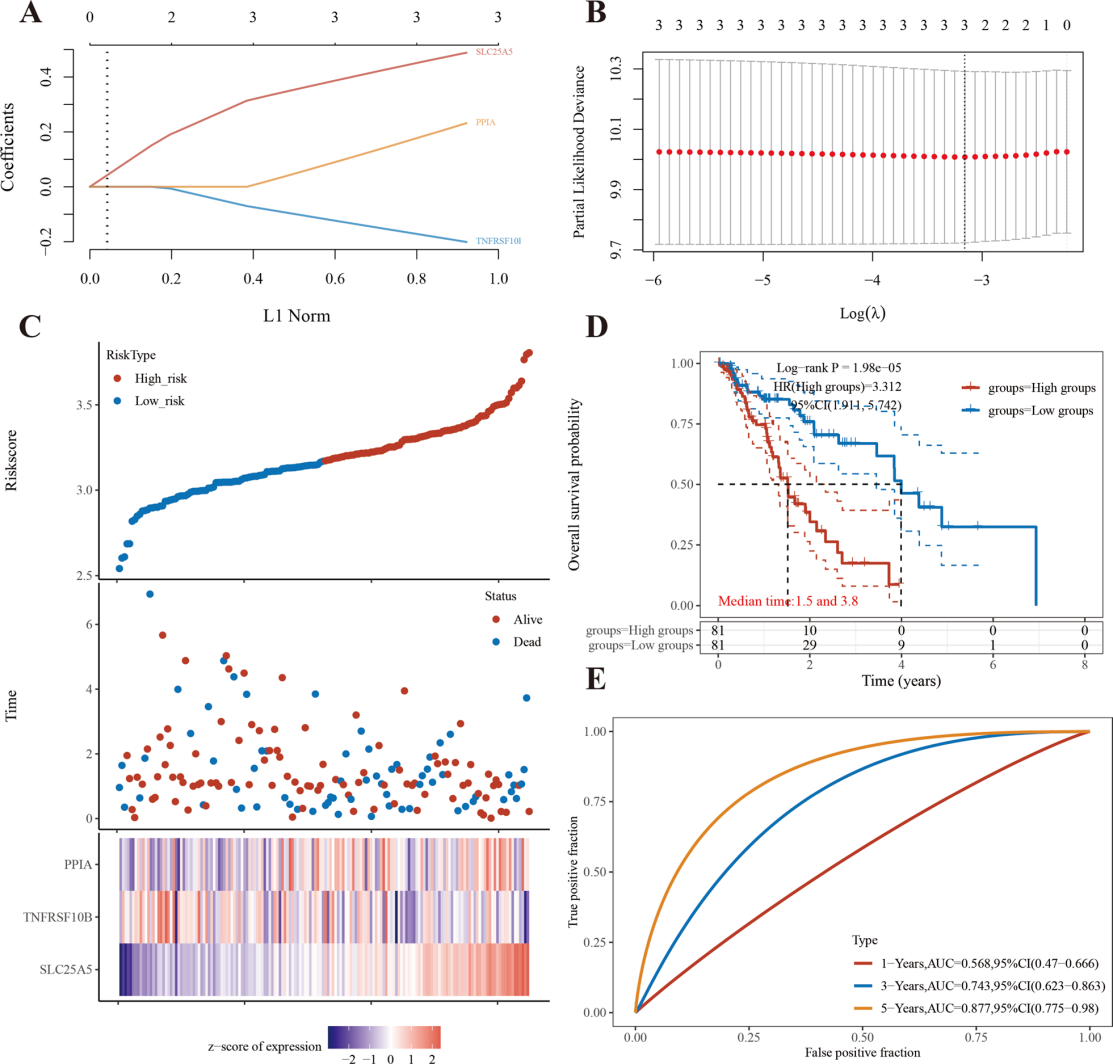
Establishment of a prognostic NRG model. **A** LASSO coefficient profiles of three NRGs. **B** Plots of the ten-fold cross-validation error rates. **C** Distribution of riskscore, survival status, and the expression of three prognostic NRGs in NRGs. **D** Overall survival curves for ESCA patients in the high-/low-risk group. **E** The ROC curve of measuring the predictive value. NRGs, necroptosis-related genes; ESCA, Esophageal carcinoma; LASSO, least absolute shrinkage and selection operator; ROC, receiver operating characteristic

After incorporating the result of the LASSO regression analysis, the corresponding three genes were selected for the signature; the model fitted the data well, and the penalty coefficient was three (Fig. 6A, B). Subsequently, we performed multivariate Cox regression analysis of the three NRGs. The results showed that these three NRGs could act as prognostic predictors when coupled with the beta value of the multivariate Cox regression. The risk score was calculated as, risk score = (0.3481) * *SLC25A5* + (− 0.0405) * *PPIA* + (− 0.0948) * *TNFRSF10B.* Based on the risk score, ESCA patients were classified into a high-risk group and a low-risk group. Compared to the low-risk group, the high-risk group had higher mortality and worse prognosis (Fig. 6C). As shown in Fig. 6D, ESCA patients belonging to the high-risk group had a higher probability of death than those belonging to the low-risk group (median time = 1.5 years vs. 3.8 years, *p* = 0.0000198). The AUCs of the risk assessment model for the three NRGs were 0.568, 0.743, and 0.877 at 1-year, 3-year, and 5-year, respectively (Fig. 6E). The model exhibited good accuracy of prediction.

### Building and validation of the predictive nomogram

**Fig. 7:**
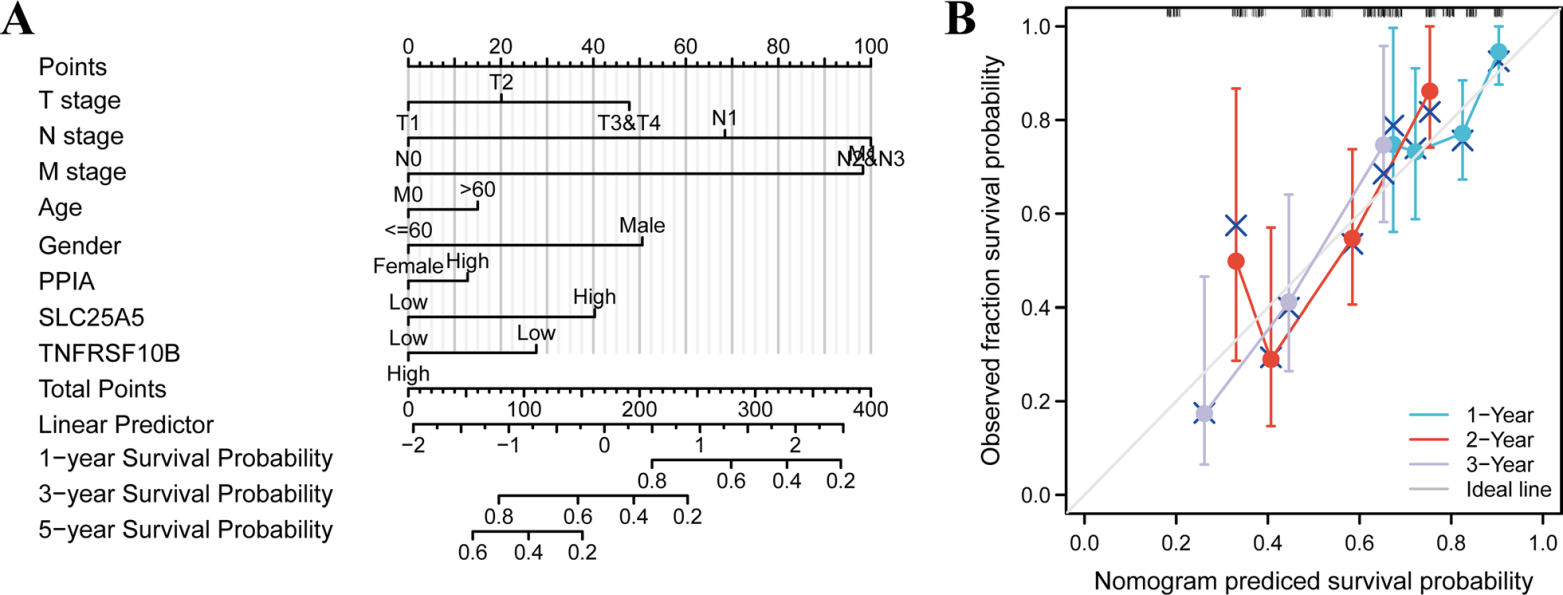
Construction of a predictive nomogram. **A** Nomogram to predict the 1-year, 3-year, and 5-year overall survival rate of ESCA patients. **B** Calibration curve for the overall survival nomogram model in the 1-year, 2- year,3-year group. A dashed diagonal line represents the ideal nomogram. ESCA, Esophageal carcinoma

Based on the three NRGs and the clinical factors (TNM stage, age, and gender), we constructed a nomogram to predict the survival probability of ESCA patients. Univariate analysis showed that N1-3, M1, and the expression of *SLC25A5* and *TNFRSF10B* were associated with the prognosis of ESCA patients (Additional file 4: Table S3). Multivariate Cox analysis indicated that N1-3 and M1 were independent predictors of prognosis (Additional file 4: Table S3). Based on the Cox regression algorithm, we produced a nomogram to predict the 1-year, 2-year, and 5-year OS rates (Fig. 7A). The calibration plots showed that the nomogram had the best prediction accuracy for 1- year and 3-year OS rates in the entire cohort (Fig. 7B).

### Validation of the predictive model

**Fig. 8:**
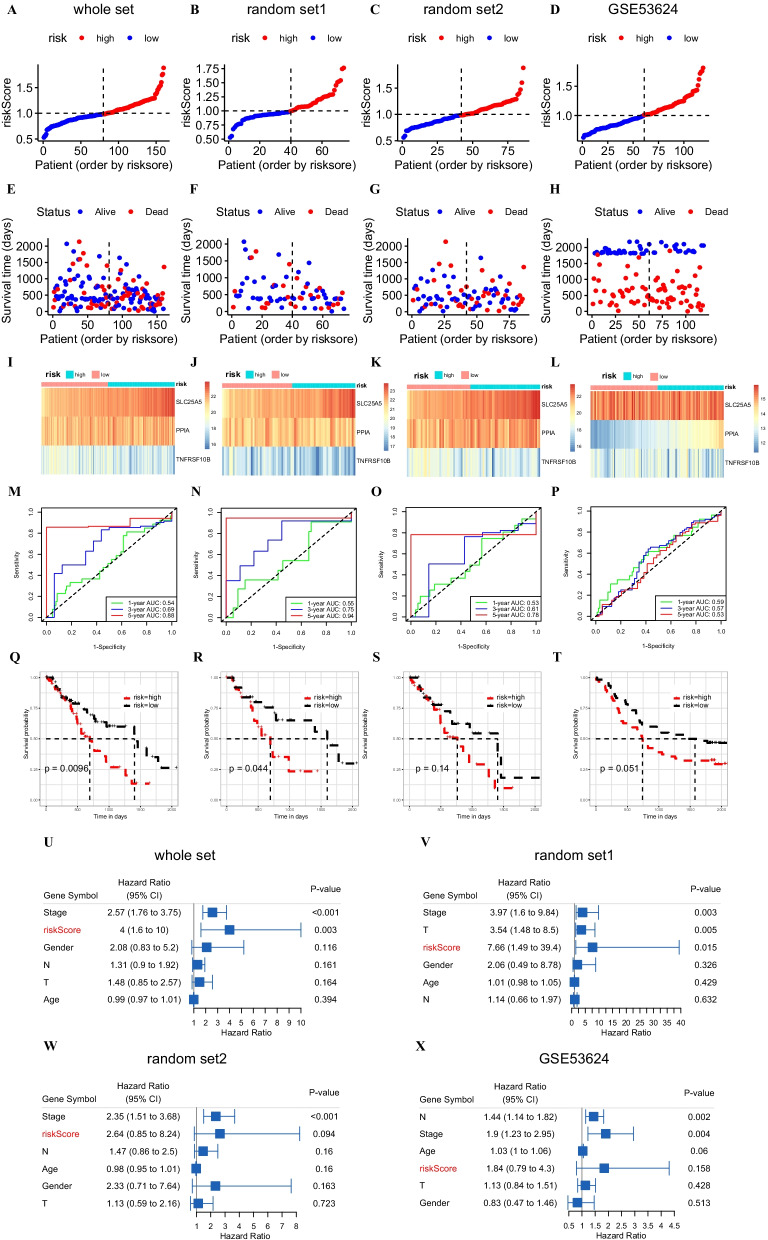
The results of various methods to verify the performance of the model based on the whole set, random set1, random set2 and GSE53624 datasets. **A**–**H** Risk score and survival time plots. **I**–**L** Expression heat map of three NRGs. **M**–**P** 1-, 3-, and 5-year ROC plot. **Q**–**T** Kaplan–Meier survival plot. **U**–**X** Forest plots for univariate Cox regression. NRGs, Necroptosis-related gene; ROC, receiver operating characteristic

To verify the applicability and prognostic value of our predictive model, we randomly divided the ESCA data set from TCGA into two equal groups (named random set1 and random set2). We used the TCGA set, random set1, random set2, and the external dataset GSE53624 for assessing the predictive ability of our model. Considering the median risk score as the cutoff value, ESCA patients were classified into a high-risk group and a low-risk group. Figure 8A–H shows the distribution of risk scores and OS status of the four sets. The heat map indicated the association between the three NRGs and the risk scores (Fig. 8I–L). The expression of the three genes differed between the tissues of high-risk and low-risk groups. Furthermore, the receiver operating characteristic (ROC) curve proved the high prediction accuracy of the model for 1-, 3-, and 5-year survival in the four datasets (AUC values were > 0.5 for all). It is noteworthy that the AUC values of the model in the whole set, random set1, and random set2 for predicting 5-year survival were greater than 0.75 (Fig. 8). The prediction performance of the model in the external dataset GSE53624 was also better (Fig. 8). In addition, the results of the Kaplan–Meier plot revealed that the high-risk group had a worse OS compared with that of the low-risk group in the whole set (*p* = 0.0096) (Fig. 8Q) and random set1 (*p* = 0.0044) (Fig. 8R). Moreover, the assessment of the correlation between prognosis and the clinical factors (T stage, N stage, stage, age, gender, and risk score) by univariate Cox and multivariate Cox regression analysis revealed that stage I and different risk scores were associated with prognosis of ESCA patients (*p* < 0.05) in the whole set and random set1 (Fig. 8U, V). However, these findings were not the same as the results obtained for the random set2 and GSE53624 dataset (*p* > 0.05) (Fig. 8W, X). Overall, the model had a good predictive effect.

### Subgroups analysis of clinical features in the predictive model

**Fig. 9:**
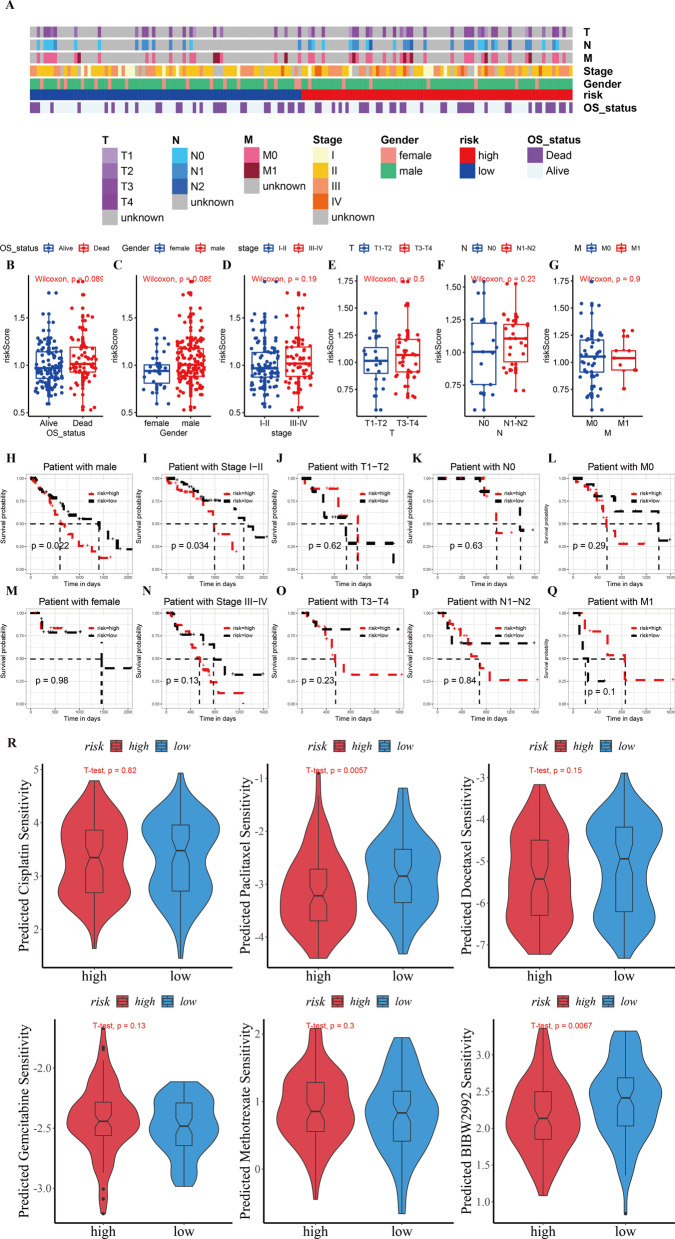
Detailed analyses of clinical data. **A** The distribution of clinical characteristics and risk for each data sample. **B–G** Differences in risk scores of patients with different clinical characteristics. **H–Q** Kaplan–Meier survival plots for different groups by clinical feature. **R** The sensitivity of six chemotherapy and target drugs in the high- and low-risk groups

Next, we compared the risk scores among the subgroups of different clinical features (T stage, N stage, M stage, tumor stage, gender, and survival). Clinical features and risks for each patient are summarized in the heat map (Fig. 9A). Unfortunately, no difference between the risker score assessment between the subgroups was observed (*p* > 0.05) (Fig. 9B–G). Meanwhile, we found that the model exhibited good OS predictive ability in the male subgroup and stage I–II subgroup (*p* < 0.05) (Fig. 9H–Q).

### Correlation between prognostic model and drug sensitivity

A combination of targeted drugs and chemotherapy is commonly used in the treatment of advanced ESCA. Consequently, we assessed the difference in efficacy of targeted drugs and chemotherapy drugs between the high-risk group and low-risk group. We predicted the efficacy of five chemotherapeutic drugs (Cisplatin, Paclitaxel, Docetaxel, Gemcitabine, and Methotrexate) and BIBW2992 against ESCA. The results indicated that the low-risk group was more sensitive to Paclitaxel and BIBW2992 than the high-risk group (*p* < 0.05) (Fig. 9R).

### Protein expression analysis of the three NRGs in ESCA

**Fig. 10:**
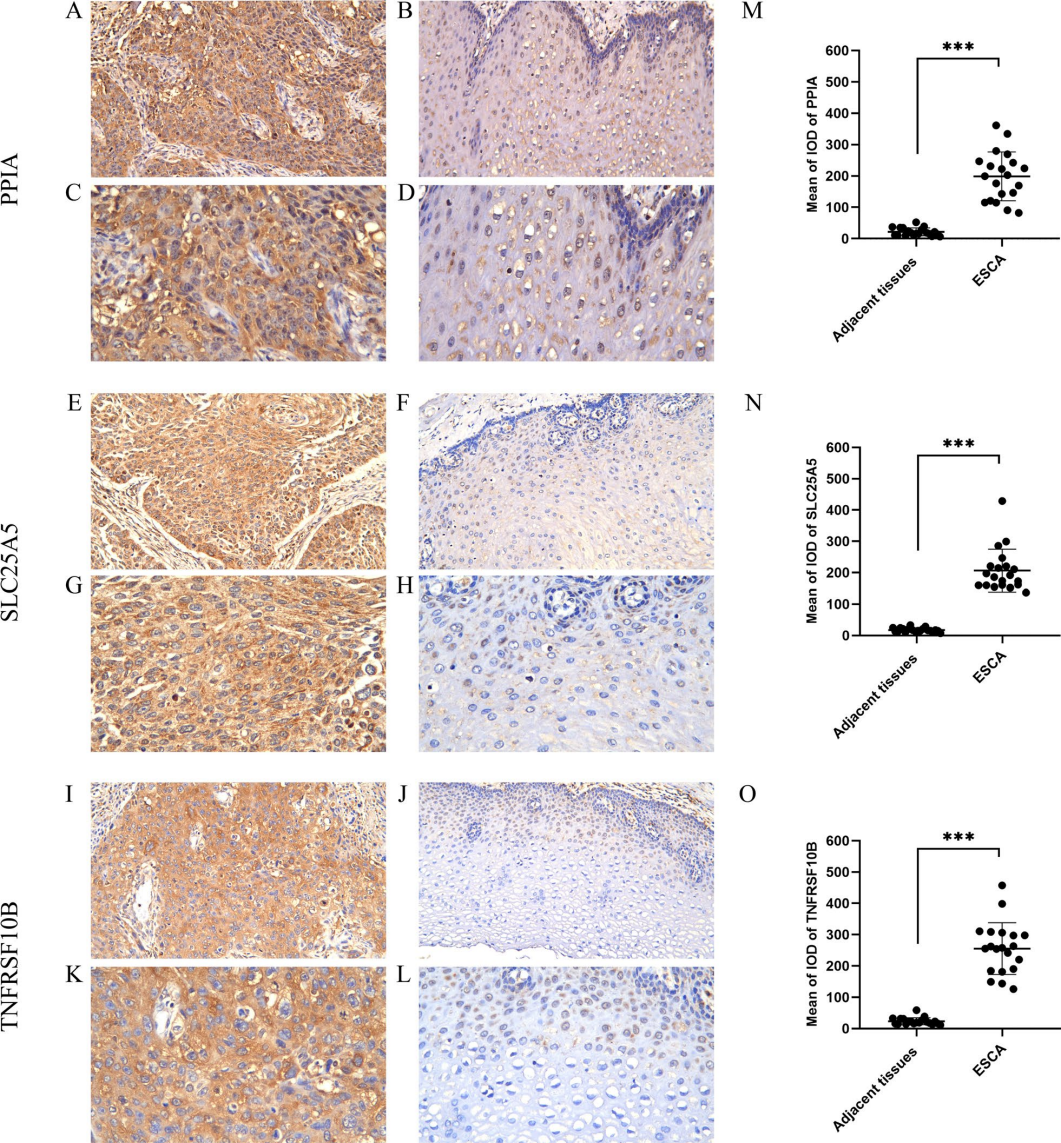
Three NGRs protein expression analysis. **A** PPIA protein expression in ESCA tumor tissue (×200 magnification); **B** PPIA protein expression in normal esophageal tissue (×200 magnification); **C** PPIA protein expression in ESCA tumor tissue (×400 magnification); **D** PPIA protein expression in normal esophageal tissue (×400 magnification); **E** SLC25A5 protein expression in ESCA tumor tissue (×200 magnification); **F** SLC25A5 protein expression in normal esophageal tissue (×200 magnification); **G** SLC25A5 protein expression in ESCA tumor tissue (×400 magnification); **H** SLC25A5 protein expression in normal esophageal tissue (×400 magnification); **I** TNFRSF10B protein expression in ESCA tumor tissue (×200 magnification); **J** TNFRSF10B protein expression in normal esophageal tissue (×200 magnification); **K** TNFRSF10B protein expression in ESCA tumor tissue (×400 magnification); **L** TNFRSF10B protein expression in normal esophageal tissue (×400 magnification); **M** Quantification of immunostains for PPIA by IOD analysis; **N** Quantification of immunostains for SLC25A5 by IOD analysis; ** O** Quantification of immunostains for TNFRSF10B by IOD analysis.***p < 0.001. ESCA, Esophageal carcinoma

We employed IHC to validate the expression of the proteins of the three NRGs in 20 pairs of ESCA tumor tissues and corresponding adjacent normal tissues. According to IHC staining analysis, the protein products of the three NRGs were located primarily in the cytoplasm, membrane, and mitochondria of cancer cells, with brown staining reflecting positive staining (Fig. 10A, C, E, G, I, K). In normal tissues, the proteins of three NRGs were only weakly expressed or not expressed (Fig. 10B, D, F, H, J, L). Based on statistical analysis, these three NRGs were expressed significantly higher in ESCA tissues than in adjacent non-tumor tissues (*P* < 0.001) (Fig. 10M–O).

### NRGs are associated with tumor immune infiltration and immune checkpoints in ESCA

**Fig. 11:**
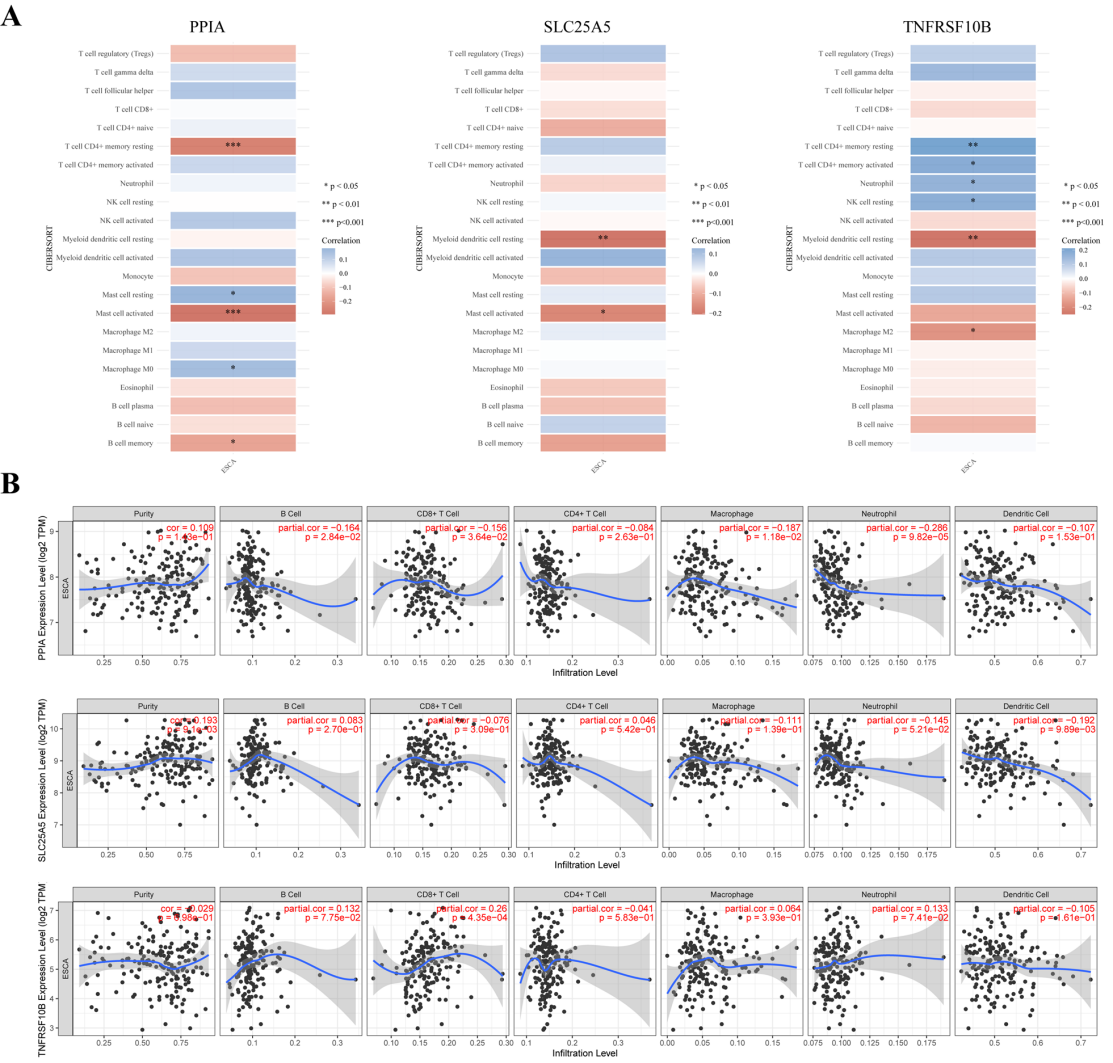
**A** The correlations between three genes and the abundance of immune cells infiltration in prognostic model. Blue color represents positive correlation, red color represents negative correlation. **B** The relationship between three prognostic NRGs expression levels and immune cell infiltration in ESCA via TIMER database. *p < 0.05, **p < 0.01, ***p < 0.001. NRGs, necroptosis-related genes; TILs, tumor-infiltrating lymphocytes; ESCA, esophageal carcinoma

Tumor-infiltrating lymphocyte levels are an independent predictor of survival in cancers. Here, we proved that the expression of prognostic NRGs (*PPIA, SLC25A5,* and *TNFRSF10B*) correlated with immune infiltration in ESCA using the TCGA dataset. According to the CIBERSORT algorithm, we found that PPIA expression was significantly negatively associated with resting memory CD4 + T cell, activated mast cell, and memory B cell (*p* < 0.05), whereas it was significantly positively correlated with resting mast cell and macrophage M0 (*p* < 0.05) (Fig. 11A). SLC25A5 expression was significantly positively correlated with resting myeloid dendritic cell and activated mast cell (*p* < 0.05) (Fig. 11A). Meanwhile, TNFRSF10B expression was significantly positively correlated with resting memory CD4 + T cell, activated memory CD4 + T cell, neutrophil, and resting NK cell, and was significantly negatively correlated with resting myeloid dendritic cell and macrophage M2 (*p* < 0.05) (Fig. 11A).

**Fig. 12:**
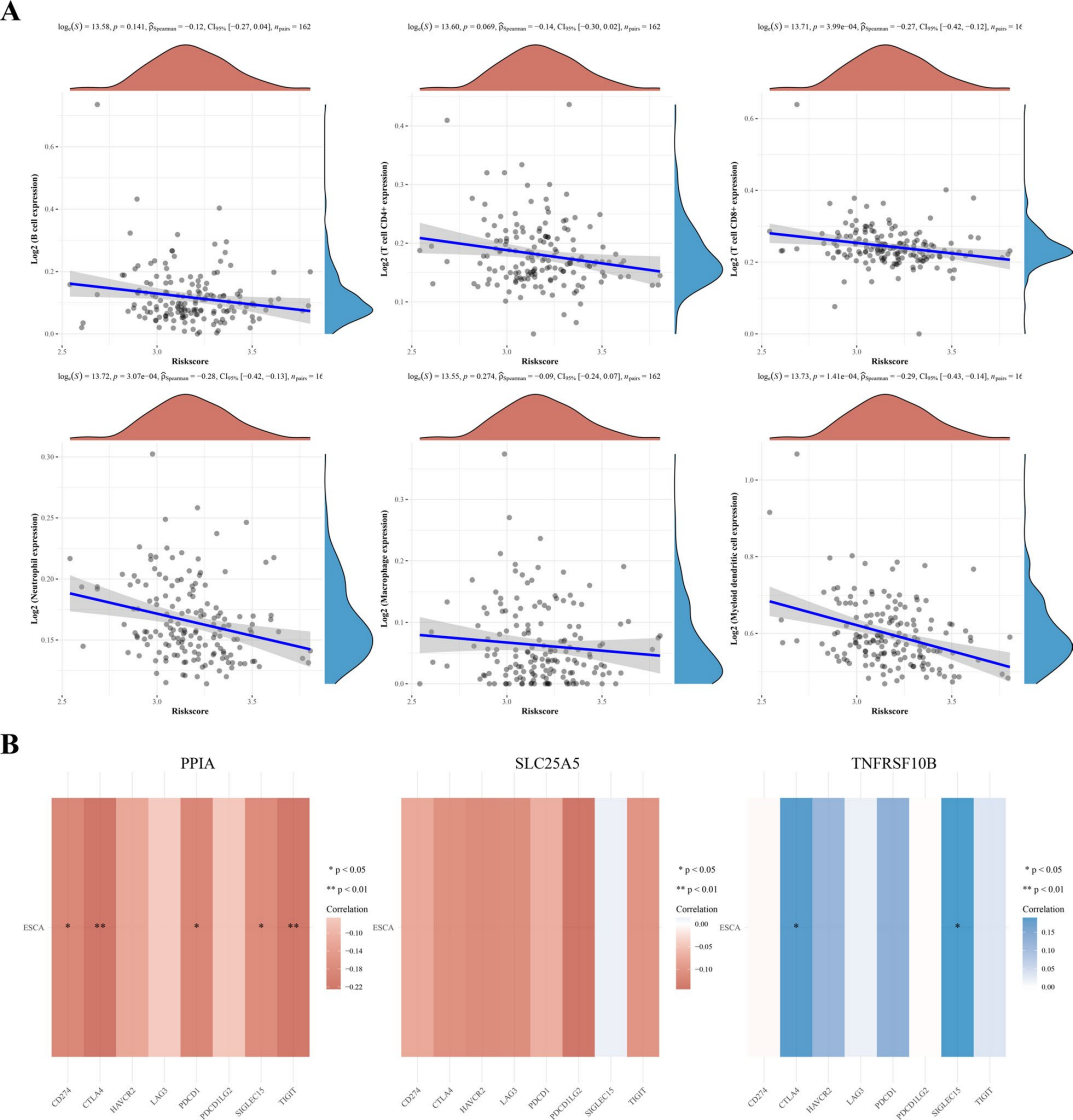
**A** The correlation between NRGs risk score and immune cell types. **B** The correlation between three NRGs and checkpoint. Blue color represents positive correlation, red color represents negative correlation. *p < 0.05, **p < 0.01. NRGs, necroptosis-related genes

To further confirm the results, we investigated the correlation between these three NRGs and immune infiltration cells in ESCA via the TIMER database. The results revealed a negative correlation between PPIA expression and the infiltration of B cells (*p* = 0.0284), CD8 + T cells (*p* = 0.0364), macrophages (*p* = 0.0118), and neutrophils (*p* = 0.0000982), whereas there was no correlation with tumor purity, CD4 + T cells, and dendritic cells (Fig. 11B). Furthermore, there was a negative correlation between SLC25A5 expression and the immune infiltration level of dendritic cells (Fig. 11B, p = 0.00989). However, SLC25A5 expression was not associated with B cells, CD4 + T cells, CD8 + T cells, neutrophils, and macrophages. Moreover, TNFRSF10B expression was positively associated with the infiltration of CD8 + T cells (Fig. 11B, p = 0.000435), while there was no association between TNFRSF10B levels and tumor purity, B cell, CD4 + T cell, macrophages, neutrophils, and dendritic cells (Fig. 11B). We further analyzed the correlation between the risk score of NRGs and immune infiltration in ESCA. As expected, the risk score of NRGs was significantly negatively correlated with the abundance of CD8 + T cells (*p* = 3.99e−4), neutrophils (*p* = 3.07e− 4), and myeloid dendritic cells (*p* = 1.141e −4) (Fig. 12A).

SIGLEC15, PDCD1LG2(PD-L2), TIGIT, PDCD1(PD-1), CD274(PD-L1), CTLA4, LAG3, and HAVCR2(TIM3) are immunological checkpoints that perform a vital function in tumor immune evasion. Considering that these three NRGs might be the predictive biomarkers in ESCA, the relationship of these NRGs with the above-mentioned checkpoints was assessed. Notably, the expression of TNFRSF10B was positively correlated with that of CTLA4 and SIGLEC15 (*p* < 0.05), while PPIA expression was negatively correlated with that of CD274, CTLA4, PDCD1, SIGLEC15, and TIGIT in ESCA (*p* < 0.05) (Fig. 12B).

## Discussion

Necroptosis is a newly discovered mechanism of programmed necrosis that plays an essential function in various cancers. Necroptosis is also the cell death mechanism in tumor cells resistant to apoptosis and is triggered by a multitude of different stimuli. The classical necroptosis pathway mediated by kinase-dependent RIPs is triggered when pro-apoptotic molecules fail to stimulate apoptotic bodies [17]. Tumor multi-drug resistance characterized by apoptosis limits the clinical application of apoptosis inducers. Therefore, targeting necroptosis may be a novel strategy to bypass apoptotic resistance and eliminate cancer cells [18]. Furthermore, the role of inflammation and immunity in necroptosis cannot be ignored. Biomarkers of necroptosis are significantly associated with the immunological profile in ESCA [19]. It has been reported that necroptosis induced by chemotherapy can stimulate inflammatory responses and elicit immunogenic and anticancer effects [20]. Necrotic tumor cells can hinder anti-tumor immunity; these cells, which are cleared by monocytes, neutrophils, and macrophages, can induce the release of inflammatory factors and ultimately trigger an adaptive immune response [21]. The critical role of NRGs in the pathogenesis of cancer makes them a potential prognostic and therapeutic target in cancer. However, the clinical significance of NRGs in ESCA has not been elucidated to date and further study is still needed.

Thus, to investigate the role of NRGs in ESCA, we used the TCGA database to analyze the expression of 52 NRGs in ESCA and normal esophageal tissues. GO and KEGG functional enrichment analyses showed that these NRGs were mostly associated with the regulation of necroptosis, Influenza A, apoptosis, NOD-like receptor signaling pathway, and NF-Kappa B signaling pathway. In addition, immune-related functions were also enriched, such as cytokine-cytokine receptor interaction, T cell activation, and positive regulation of cell adhesion. As expected, the enriched functions were associated with the tumor immune microenvironment, carcinogenesis, progression, and necroptosis in ESCA. Preliminary research has indicated that NOD-like receptor signaling pathways are possible predictors of esophageal adenocarcinoma [22].

Furthermore, our results indicated that three differentially expressed NRGs (*SLC25A5, PPIA,* and *TNFRSF10B*) were significantly related to the prognosis of ESCA. *TNFRSF10B* was found to be a favorable prognostic gene, while *SLC25A5* and *PPIA* were related to adverse prognosis in ESCA. We then performed LASSO Cox regression to construct a prognostic signature based on the three prognostic NRGs. According to the risk score, ESCA patients were divided into two groups (high-risk and low-risk groups), and the results revealed that the high-risk group had a significantly poorer OS than the low-risk group. Univariate analysis indicated that N1-3, M1, and high expression of SLC25A5 and TNFRSF10B were correlated with the prognosis of ESCA patients (Additional file 4: Table S3). Furthermore, according to Multivariate Cox analysis, N1-3 and M1 were independent predictors of prognosis. The ROC curve confirmed the prognostic signature to be an independent prognostic indicator by showing that it could predict the 1-year, 3-year, and 5-year OS rates relatively well compared with an ideal model in the entire cohort.

To test the applicability and prognostic value of our model, we randomly divided the ESCA data set from TCGA into random set1 and random set2. We used random set1, random set2, and the external dataset GSE53624 for assessing the predictive ability of our model. The model exhibited a good predictive effect in all three datasets. Subgroups analysis of clinical features in the predictive model indicated that the model had good OS predictive ability in the male subgroup and stage I-II subgroup. Correlation between the prognostic model and clinical treatment indicated that the low-risk group was more sensitive to Paclitaxel and BIBW2992 than the high-risk group. Subsequently, IHC staining analysis showed the three NRGs were expressed significantly higher in ESCA tissues than in adjacent non-tumor tissues. In summary, we, for the first time, constructed a necroptosis‑related prognostic gene signature for ESCA, which provides new clues for prognostic prediction in ESCA patients.

TNFRSF10B is a member of the TNF-receptor superfamily that is activated by TNF-related apoptosis-inducing ligand (TRAIL). TNFRSF10B/DR5 delivers apoptotic signals to the cell and induces apoptosis in cancer cells [23]. The expression level of TNFRSF10B was associated with tumor progression and apoptosis [24]. Furthermore, the high expression of DR5 mediated the extrinsic apoptotic pathway in various cancer cells [25]. However, He et al. showed that the low expression of TNFRSF10B was associated with a poor prognosis in esophageal squamous cell carcinoma [26, 27], which is consistent with our findings. SLC25A5 (ANT2) is a by-product of nucleotide transferase which is specifically expressed in proliferating cells and participates in glycolytic metabolism. Hence, SLC25A5 is associated with cell growth and differentiation [28]. Depletion of SLC25A5 can cause mitochondrial dysfunction and induce oxidative stress, leading to erythrocyte anemia and B-cell depletion [29]. SLC25A5 promotes apoptosis through the regulation of bcl-2, caspase-3, and bax in prostate cancer [30]. Studies have shown that high expression of SLC25A5 in cervical cancer could be an independent prognostic factor [31]. PPIA (Cyclophilin A) is a member of the immunophilin family and acts as an immune inflammatory mediator that secretes oxidative stress-induced, which promotes the formation of foam cells by increasing the levels of ROS and pro-inflammatory cytokines. Furthermore, PPIA is involved in biological processes such as intracellular signaling, transcription, and apoptosis, therefore playing critical roles in microorganismal infections, inflammatory diseases, and tumor proliferation [32–34]. An increase in PPIA levels may lead to macrophage apoptosis through activation of mitochondrial death signaling pathways and caspase 3 cascade [35]. Studies have confirmed that the upregulation of PPIA is associated with a poor OS in diseases such as lung adenocarcinoma [36]. Some researchers have indicated that overexpression of PPIA is associated with decreased survival in esophageal squamous cell carcinoma and shown it to be an independent prognostic factor [37, 38]. Although these studies reveal the relationship between the NRGs and tumor progression, none of these studies have explored the expression and function of the NRGs in ESCA.

The tumor immune microenvironment is mainly composed of tumor-infiltrating lymphocytes and other immune cells such as dendritic cells, neutrophils, and macrophages. Tumor-infiltrating lymphocytes can inhibit or promote tumor progression [39]. Our study confirmed that the expression levels of the three NRGs were significantly associated with immune cell infiltration in ESCA. Early findings suggested that abundant CD8+ T cells and CD4+ T cells could be prognostic indicators for the clinical outcome in cancers [40, 41]. Our results are consistent with the previous reports suggesting that TNFRSF10B expression associated with high CD8+ T cell abundance may indicate better clinical outcomes. Preliminary research revealed that the tumor microenvironment is associated with tumor growth and prognosis in ESCA, and increasing levels of immune infiltrates reduce the risk of distant metastasis and death [27, 42]. Our results showed that the expression levels of the three prognostic NRGs were significantly correlated with that of immunological checkpoints in ESCA. Our research might provide more clues for immunotherapy strategies in ESCA. All these outcomes illustrate that tumor immune evasion and antitumor immunity might be implicated in the three prognostic NRGs-mediated carcinogenic processes in ESCA.

It was interesting to note that different approaches yielded inconsistent results regarding the relationship between immune cells infiltration and the three prognostic NRGs. This inconsistency may be attributable to the following reasons. Although flow cytometry, immunohistochemistry staining, or single-cell sequencing can be used to estimate the immune cell status in a tumor sample, each has limitations that prevent them from being widely applied. Therefore, we used computational methods to evaluate immune-cell composition from bulk RNA-sequencing data. First of all, between the the actual situation and computer-based algorithms, there were some variations. Next, the mechanisms of immune cell infiltration in tumors are complex, and they are inevitably affected by intratumorally heterogeneity and small sample sizes. In the end, various algorithms are used in these methods, and they all have advantages and disadvantages.

Although the prognostic model exhibited good performance in the TCGA database and external datasets, our study still has some limitations in lacking mechanistic experiment and further experimental validation are needed.

## Conclusion

In summary, we identified three prognosis-associated NRGs (*SLC25A5, PPIA,* and *TNFRSF10B*) and constructed a novel necroptosis-related prognostic gene signature. Furthermore, in this study, we demonstrated that *SLC25A5, PPIA,* and *TNFRSF10B* were correlated with tumor immune microenvironment infiltration and may be potential prognostic biomarkers for ESCA. The prognostic model exhibited good performance for the prediction of ESCA prognosis in TCGA database and external datasets. Our findings will benefit the treatment and diagnosis of ESCA. Nevertheless, the study results need to be validated in future fundamental research and extensive clinical trials.

## Materials and methods

### Datasets and data processing

The UCSC Xena database was utilized to obtain TCGA-ESCA RNA-Seq FPKM data and clinical information and survival information (https://toil-xena-hub.s3.us-east-1.amazonaws.com/download/TcgaTargetGtex_rsem_gene_tpm.gz; Full metadata) [43]. One independent ESCA gene expression profiles (GSE53624) were downloaded from the Gene Expression Omnibus (GEO) database (https://www.ncbi.nlm.nih.gov/geo/) and processed for analysis [44]. There are 162 ESCA and 11 normal cases with clinical data were downloaded from the database (Table 1). 159 NRGs were downloaded from Kyoto Encyclopedia of Genes and Genomes (KEGG) database (Additional file 1: Table S1) [45–47]. All statistical analyses were carried out using R version v4.0.3.

**Table 1:** Clinical characteristics of patients with EC

Characteristic	Levels	Overall
n		161
T stage, n (%)	T1	27 (18.6%)
	T2	37 (25.5%)
	T3	77 (53.1%)
	T4	4 (2.8%)
N stage, n (%)	N0	66 (45.8%)
	N1	63 (43.8%)
	N2	9 (6.2%)
	N3	6 (4.2%)
M stage, n (%)	M0	121 (93.8%)
	M1	8 (6.2%)
Pathologic stage, n (%)	Stage I	16 (11.3%)
	Stage II	69 (48.6%)
	Stage III	49 (34.5%)
	Stage IV	8 (5.6%)
Radiation therapy, n (%)	No	107 (74.8%)
	Yes	36 (25.2%)
Primary therapy outcome, n (%)	PD	9 (9.7%)
	SD	7 (7.5%)
	PR	3 (3.2%)
	CR	74 (79.6%)
Gender, n (%)	Female	23 (14.3%)
	Male	138 (85.7%)
Race, n (%)	Asian	38 (26.6%)
	Black or African American	5 (3.5%)
	White	100 (69.9%)
Age, n (%)	≤ 60	82 (50.9%)
	> 60	79 (49.1%)
Weight, n (%)	≤ 70	76 (47.8%)
	> 70	83 (52.2%)
Height, n (%)	< 170	47 (30.9%)
	≥ 170	105 (69.1%)
BMI, n (%)	≤ 25	84 (55.3%)
	> 25	68 (44.7%)
Histological type, n (%)	Adenocarcinoma	80 (49.7%)
	Squamous cell carcinoma	81 (50.3%)
Histologic grade, n (%)	G1	16 (12.7%)
	G2	66 (52.4%)
	G3	44 (34.9%)
Residual tumor, n (%)	R0	121 (90.3%)
	R1	11 (8.2%)
	R2	2 (1.5%)
Smoker, n (%)	No	47 (32.9%)
	Yes	96 (67.1%)
Alcohol history, n (%)	No	46 (29.1%)
	Yes	112 (70.9%)
OS event, n (%)	Alive	97 (60.2%)
	Dead	64 (39.8%)
DSS event, n (%)	Alive	115 (71.9%)
	Dead	45 (28.1%)
PFI event, n (%)	Alive	83 (51.6%)
	Dead	78 (48.4%)
Age, median (IQR)		60 (53, 72)

### NRGs differential expression analysis

A total of 52 NRGs were extracted and showed in Additional file 2: Table S2, which was the most representative significance genes. The "limma" software packages were used to identify the differences in NRG expression between ESCA and normal esophageal tissues, and |Log2-fold change |> 1 and *p*-values < 0.05 were set as the filter conditions. Analyzing the mutation rates of 52 NRGs in ESCA patients and mapping waterfall and frequency plots of 52 NRGs in ESCA patients were generated using the "maftools" software package [48].

### Functional enrichment analysis

Gene Ontology (GO) analyses, including biological processes, molecular functions, and cellular components, and KEGG pathway enrichment analysis were used to identify characteristic biological attributes via the R package “ClusterProfiler [49]”. Data visualization was carried out using R package "ggplot2 [50]". *p* < 0.05 was considered to be statistically significant.

### Establishment of necroptosis risk scoring prognosis signature

The correlation between NRGs expression and survival was analyzed using univariate survival analysis. The Kaplan–Meier [51] was used to compare the survival rates of different NRGs expression. The risk score was calculated from the formula of the regression coefficients in the multiple Cox regression model. Thereafter we established the prognostic model for three prognostic NRGs by LASSO Cox regression analysis [52]. According to the median risk score, ESCA patients were divided into two subgroups (high-risk and low-risk), then we compared OS between two subgroups by KM analysis. Time ROC analysis was employed to predict accuracy of the risk score. Based on the clinical characteristics (TNM stage, gender, age and three NRGs), we constructed a nomogram to quantitatively predict 1-, 3-, and 5-year OS. Each variable such as *p*-value, HR and 95% CI were visualized by a forest by R package "forestplot".

### Validation of predictive model

We used TCGA ESCA database with external ESCA dataset GSE53624 to assess and verify the predictive ability of our prognostic model [53, 54]. The Clinical and survival information of the whole set was derived from the TCGA dataset, and the external dataset GSE53624 was obtained from the GEO database through the GEO query R package. TCGA was equally divided into two groups by random sampling, named Random set1, 2. Time ROC analysis was employed to predict accuracy of 1-, 3-, and 5-year survival. The correlation between risk score and survival time was examined via KM survival analysis. Prognostic and predictive value of Risk score was validated by Univariate Cox analysis. All analytical analyses were performed using the R packages "ggplot2", "coxph function", "pheatmap", "timeROC", "survival", and "survminer".

### Correlation between prognostic model and drug sensitivity

We used the R package “pRRophetic” to predict drug sensitivity and the results were visualized by the expression matrix.

### Immunohistochemical staining

In order to further validate the expression of the three NRGs between ESCA and adjacent normal tissues, 20 paired ESCA tissues from Liuzhou People's Hospital were collected for immunohistochemical staining. The study was approved by the Ethics Committee of Liuzhou People's Hospital (Reference No. KY2021-026-01) and conducted according to the Declaration of Helsinki. Formalin fixed paraffin-embedded tissue were analyzed by Immunohistochemistry with PPIA antibody (1:100; Proteintech, China), SLC25A5(1:100; Proteintech, China), and TNFRSF10B (1:150; OriGene China) and horseradish peroxidase conjugated secondary antibodies (Maxim, China). For IHC quantification, the integrated optical density (IOD) for each slice was calculated using the Image-ProPlus6.0 software (Media Cybernetics, USA).

### Tumor microenvironment estimation

We used CIBERSORT algorithm to confirm the association between three NRGs and the abundance of 22 infiltrating immune cells. Then we used spearman correlation analysis to further confirm the association between the risk scores and those NRGs expression. This study also explored the correlation between three NRGs and 8 immune checkpoint molecules. All statistical analyses information mentioned were visualized via R version 4.0.3.

### Tumor immune estimation resource (TIMER) database

TIMER (https://cistrome.shinyapps.io/timer/) dataset comprise six tumor-infiltrating immune subsets. Calculated the levels of six subgroups for 10,897 tumors in 32 cancers using the TCGA. Based on the database, gene expression and tumor immune infiltration (B cells, CD4+ T cells, CD8+ T cells, Dendritic cells, Macrophages, and Neutrophils) were analyzed in several cancer types. Using the TIMER dataset, we examined the mRNA expression of these prognostic NRGs in patients with ESCA.

### Statistical analysis

All Statistical analysis analyzed were conducted by the Log-rank test, such as fold-change (FC), HR, and *p-*values. The correlation between particular variables was validated using the Spearman's correlation analysis or Pearson correlation analysis. *p*-value or Log-rank *p*-value of < 0.05 was considered as having statistical significance.

## Supplementary Information

**Additional file 1: Table S1**. 159 NRGs from KEGG in EC.

**Additional file 2: Table S2**. 52 NRGs among the DEGs between EC and normal samples.

**Additional file 3: Figure S1**. Univariate survival analysis of differentially expressed NRGs with prognostic value in ESCA.

**Additional file 4: Table S3**. Analysis of factors affecting the prognosis of patients with esophageal carcinoma.

## Data Availability

The datasets generated and analysed during the current study are available in the [UCSC Xena database] repository, https://xenabrowser.net/.

## Abbreviations

ESCA: Esophageal carcinoma
NRGs: Necroptosis-related genes
DEGs: Differentially expressed genes
UCSC Xena: University Of Cingifornia Sisha Cruz
TCGA: The Cancer Genome Atlas
KEGG: Kyoto Encyclopedia of Genes and Genomes
GO: Gene ontology
LASSO: Least absolute shrinkage and selection operator
OS: Overall survival
ROC: Receiver operating characteristic
TNFR1: Tumor necrosis receptor 1
TLRs: Toll-like receptors
RIPs: Receptor interacting proteins
RIPK1/RIPK3: Receptor-interacting serine/threonine protein kinase 1/3
MLKL/pMLKL: Mixed lineage kinase domain-like protein
ROS: Reactive oxygen species
TIMER: Tumor Immune Estimation Resource
KM plotter: Kaplan–Meier plotter
STRING: Search Tool for the Retrieval of Interacting Genes
FC: Fold-change
HR: Hazard ratio
BP: Biological process
CC: Cellular component
MF: Molecular function
PFS: Progression-free surviva
DSS: Disease-specific survival
AUCs: Area under the curves
NK cell: Natural killer cell
