# Supplementary figures and images for: Identification and validation of necroptosis-related prognostic gene signature and tumor immune microenvironment infiltration characterization in esophageal carcinoma
Source: BMC Gastroenterol. 2022 Jul 15;22:344. doi: 10.1186/s12876-022-02423-6 (PMC9284853; doi:10.1186/s12876-022-02423-6)

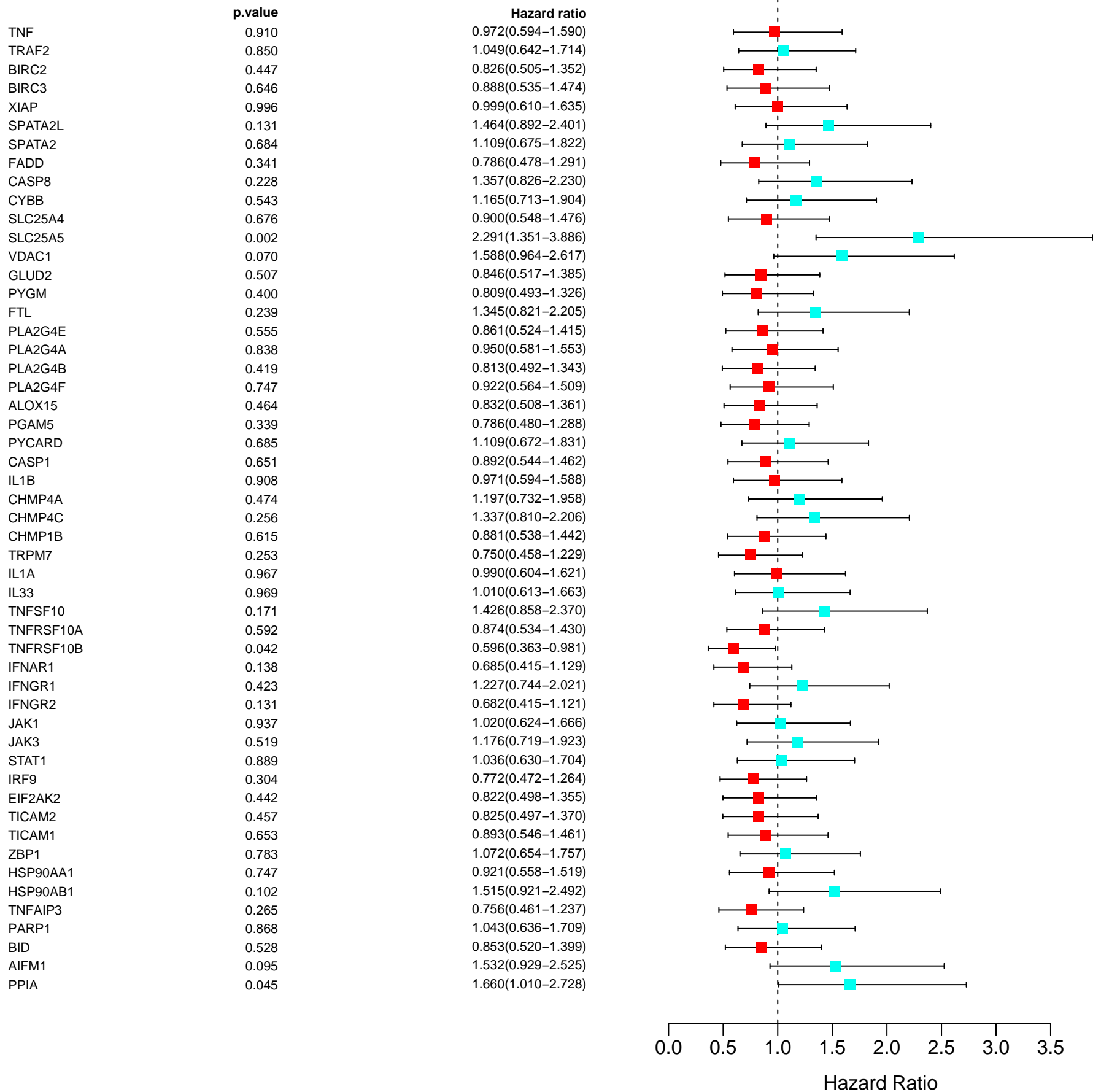

Supplement: Supplementary file 3 — Additional file 3: Figure S1. Univariate survival analysis of differentially expressed NRGs with prognostic value in ESCA. [file 12876_2022_2423_MOESM3_ESM.pdf]
