# Supplementary material for: Identification and validation of necroptosis-related prognostic gene signature and tumor immune microenvironment infiltration characterization in esophageal carcinoma
Source: BMC Gastroenterol. 2022 Jul 15;22:344. doi: 10.1186/s12876-022-02423-6 (PMC9284853; doi:10.1186/s12876-022-02423-6)
Supplement: Supplementary file 4 — Additional file 4: Table S3. Analysis of factors affecting the prognosis of patients with esophageal carcinoma. [file 12876_2022_2423_MOESM4_ESM.docx]

| Table S3 Analysis of factors affecting the prognosis of patients with esophageal carcinoma | | | | | | |
| --- | --- | --- | --- | --- | --- | --- |
| Characteristics | Total(N) | Univariate analysis | |  | Multivariate analysis | |
|  |  | Hazard ratio (95% CI) | P value |  | Hazard ratio (95% CI) | P value |
| **T stage** | 145 |  |  |  |  |  |
| T1 | 27 | Reference |  |  |  |  |
| T2 | 37 | 0.868 (0.388-1.940) | 0.729 |  |  |  |
| T3&T4 | 81 | 1.211 (0.599-2.450) | 0.594 |  |  |  |
| **N stage** | 144 |  |  |  |  |  |
| N0 | 66 | Reference |  |  |  |  |
| N1 | 63 | 2.853 (1.515-5.373) | **0.001** |  | 2.310 (1.100-4.851) | **0.027** |
| N2&N3 | 15 | 3.602 (1.483-8.745) | **0.005** |  | 3.497 (1.201-10.181) | **0.022** |
| **M stage** | 129 |  |  |  |  |  |
| M0 | 121 | Reference |  |  |  |  |
| M1 | 8 | 5.075 (2.312-11.136) | **<0.001** |  | 2.835 (1.160-6.926) | **0.022** |
| **Age** | 161 |  |  |  |  |  |
| <=60 | 82 | Reference |  |  |  |  |
| >60 | 79 | 0.855 (0.519-1.408) | 0.538 |  |  |  |
| **Gender** | 161 |  |  |  |  |  |
| Female | 23 | Reference |  |  |  |  |
| Male | 138 | 2.271 (0.907-5.686) | 0.080 |  | 1.920 (0.564-6.535) | 0.296 |
| **PPIA** | 161 |  |  |  |  |  |
| Low | 80 | Reference |  |  |  |  |
| High | 81 | 1.183 (0.720-1.945) | 0.507 |  |  |  |
| **SLC25A5** | 161 |  |  |  |  |  |
| Low | 80 | Reference |  |  |  |  |
| High | 81 | 2.433 (1.423-4.161) | **0.001** |  | 1.659 (0.833-3.305) | 0.150 |
| **TNFRSF10B** | 161 |  |  |  |  |  |
| Low | 80 | Reference |  |  |  |  |
| High | 81 | 0.536 (0.324-0.888) | **0.016** |  | 0.784 (0.413-1.489) | 0.457 |
